# Gated Silicon Drift Detector Fabricated from a Low-Cost Silicon Wafer

**DOI:** 10.3390/s150512022

**Published:** 2015-05-22

**Authors:** Hideharu Matsuura, Shungo Sakurai, Yuya Oda, Shinya Fukushima, Shohei Ishikawa, Akinobu Takeshita, Atsuki Hidaka

**Affiliations:** Department of Electrical and Electronic Engineering, Osaka Electro-Communication University, 18-8 Hatsu-cho, Neyagawa, Osaka 572-8530, Japan; E-Mails: ee11a045@oecu.jp (S.S.); ee11a022@oecu.jp (Y.O.); e09071@oecu.jp (S.F.); mf14a002@oecu.jp (S.I.); ee11a056@oecu.jp (A.T.); h-atsuki@mail.osakac.ac.jp (A.H.)

**Keywords:** gated silicon drift detector, silicon drift detector, low-cost X-ray detector, thick X-ray detector

## Abstract

Inexpensive high-resolution silicon (Si) X-ray detectors are required for on-site surveys of traces of hazardous elements in food and soil by measuring the energies and counts of X-ray fluorescence photons radially emitted from these elements. Gated silicon drift detectors (GSDDs) are much cheaper to fabricate than commercial silicon drift detectors (SDDs). However, previous GSDDs were fabricated from 10-kΩ·cm Si wafers, which are more expensive than 2-kΩ·cm Si wafers used in commercial SDDs. To fabricate cheaper portable X-ray fluorescence instruments, we investigate GSDDs formed from 2-kΩ·cm Si wafers. The thicknesses of commercial SDDs are up to 0.5 mm, which can detect photons with energies up to 27 keV, whereas we describe GSDDs that can detect photons with energies of up to 35 keV. We simulate the electric potential distributions in GSDDs with Si thicknesses of 0.5 and 1 mm at a single high reverse bias. GSDDs with one gate pattern using any resistivity Si wafer can work well for changing the reverse bias that is inversely proportional to the resistivity of the Si wafer.

## 1. Introduction

Various types of X-ray detectors, such as silicon (Si) pin detectors and silicon drift detectors (SDDs) [1–29], are used to measure the energy and photon count of X-ray fluorescence photons. Si X-ray detectors with a thick Si substrate, a large active area, and small capacitance are desirable [29–32].

A pin structure is used to collect charge carriers, the number of which are proportional to the energy of an X-ray photon. In X-ray fluorescence spectroscopy, the capacitance of a pin detector increases with the active area of the detector because the anode (n-type layer) and the cathode (p-type layer) have equal areas. The increase in the capacitance degrades its performance. However, SDDs have a much smaller capacitance than pin detectors [1]. This is because the anode, which is on one surface of the n^−^ Si substrate (n^−^ or i-layer), is much smaller than the pin detector, whereas the entrance window layer, which is the cathode on the opposite surface, is kept large [1]. The anode is surrounded by multiple p-type rings (p-rings), to which a different bias voltage is applied. The resulting electric field makes the electrons flow smoothly toward the anode. To form a sufficiently strong electric field toward the anode in the SDD, the p-rings are electrically coupled with expensive built-in metal-oxide-semiconductor field-effect transistors (MOSFETs) or implanted resistors.

To fabricate low-cost X-ray detectors, we have designed several simple-structure SDDs without MOSFETs or implanted resistors [33–43], one of which is a gated silicon drift detector (GSDD) [37,38,40–43]. In GSDDs fabricated by using a 0.625-mm-thick n^−^ Si substrate with a resistivity (ρ_Si_) of 10 kΩ·cm, an energy resolution of 145 eV at 5.9 keV was obtained from a ^55^Fe source at −38 °C [41]. The effective active area of the detector was approximately 18 mm^2^ by irradiating X-ray photons through a 0.1-mm-diameter pinhole [40].

The 10-kΩ·cm Si wafers are more expensive than the 2-kΩ·cm Si wafers used in commercial SDDs. In the present study, to fabricate much cheaper X-ray detectors, we used a device simulation to design adequate gate patterns for GSDDs formed from 2-kΩ·cm Si wafers.

## 2. Structure and Advantages of Gated Silicon Drift Detectors

**Figure 1:**
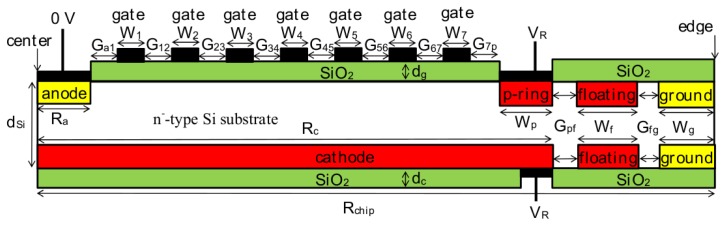
Half of a schematic cross section of a cylindrical GSDD structure with one p-ring and seven gates. The same negative voltage was applied to the cathode, the p-ring, and all the gates.

GSDDs have a cathode and only one p-ring, and to which the same reverse bias can be applied. Figure 1 shows half of a schematic cross section of a cylindrical GSDD with seven ring-shaped gates and one p-ring that does not contain MOSFETs or implanted resistors [37,38,40–43].

In SDDs and GSDDs, n-type layers (anode and ground rings) and p-type layers (cathode, p-ring, and floating rings) are fabricated by the same processes. In SDDs, multiple inner p-rings located between the anode and the p-ring are formed. Compared with GSDDs, the extra fabrication processes in SDDs are for creating the built-in MOSFETs or implanted resistors to couple the p-rings together electrically, which lowers the yield rate of detectors. The passivating oxide layers (SiO_2_) are formed, and the anode, p-ring, ground rings, and cathode are metallized. During metallization, the innermost p-ring is also metallized in SDDs, whereas gates are formed in GSDDs.

In GSDDs, no extra fabrication processes are required to form the gates because the metal gates are formed on the SiO_2_ during metallization of the anode and the p-ring. As a result, the fabrication of GSDDs is much simpler than that of commercial SDDs. Moreover, the same high reverse bias can be applied to the cathode, the p-ring, and all the gates, which means that GSDDs require only one high-voltage source. Therefore, GSDDs greatly reduce the cost of the X-ray detection system.

## 3. Device Simulation Processes

The device simulations were carried out by using the ATLAS Device Simulator (Silvaco International). All the simulations were performed by solving Poisson's equation and the carrier continuity equations. This provides a complete description of the system in terms of electrical quantities, such as electric potential and electric field distributions, carrier densities, and current densities.

The thicknesses of the n^−^ Si substrate (*d*_Si_) were 0.5 and 1 mm, and the values of ρ_Si_ were 2 and 10 k Ω·cm. The radius of the anode (*R*_a_) at the center of the cylindrical GSDD was fixed as 0.055 mm, which kept the capacitance of all GSDDs small. The widths of the p-ring (*W*_p_), p-type floating rings (*W*_f_), and n-type ground rings (*W*_g_) were 0.545, 0.03 and 0.39 mm, respectively. The gap between the p-ring and the floating ring (*G*_pf_) and the gap between the floating and ground rings (*G*_fg_) were all 0.04 mm. The thickness of SiO_2_ on the cathode (*d*_c_) was 0.75 μm. The thickness of SiO_2_ on the other side (*d*_g_) was changed to constrain the electric field in the SiO_2_ between the gates and the Si substrate at ≤ 2.5 MV/cm, which is less than the SiO_2_ breakdown electric field of 10 MV/cm [44]. The sheet density of positive fixed charges in SiO_2_ near the SiO_2_/Si interface (*Q*_F_) was fixed as 3 × 10^10^ cm^−2^, which has been reported for the present fabrication process [45]. The acceptor densities of the cathode, p-ring, and floating rings were 1 × 10^18^ cm^−3^, and the donor densities of the anode and ground rings were 1 × 10^19^ cm^−3^. The depths of the cathode, p-ring, anode, ground rings, and floating rings were all 1 μm.

Seven gates were considered in this study. Figure 1 shows that *G*_a1_ was the gap between the anode and the innermost gate, and *G*_12_, *G*_23_, *G*_34_, *G*_45_, *G*_56_ and *G*_67_ were the gaps between the gates, from the innermost to outermost. *G*_7p_ was the gap between the outermost gate and the p-ring. *W*_1_, *W*_2_, *W*_3_, *W*_4_, *W*_5_, *W*_6_ and *W*_7_ were the widths of the seven gates, from the innermost to outermost, respectively. The radii of the cathode (*R*_c_ = 3 mm) and the GSDD chip (*R*_chip_ = 3.5 mm) were fixed. As a result, the area inside the inner edge of the p-ring (*S*_area_) was 18.9 mm^2^, which is nearly equal to that of commercial small-area SDDs. The same reverse bias voltage(*V*_R_) was applied to the cathode, the p-ring, and all the gates.

## 4. Simulation Results and Discussion

### 4.1. 0.5-mm-Thick GSDD Formed from a 10-kΩ·cm Si Wafer

The *d*_Si_ and ρ_Si_ of the n^−^ Si substrate were 0.5 mm and 10 kΩ·cm, respectively, and *d*_g_ was 0.75 μm. In Gate A, the values of *W*_1_, *W*_2_, *W*_3_, *W*_4_, *W*_5_, *W*_6_, and *W*_7_ were 0.1, 0.1, 0.19, 0.29, 0.39, 0.47 and 0.51 mm, respectively. *G*_a1_ was 0.04 mm and *G*_12_ and *G*_23_ were both 0.03 mm. *G*_34_, *G*_45_, *G*_56_, *G*_67_ and *G*_7p_ were all 0.05 mm.

**Figure 2:**
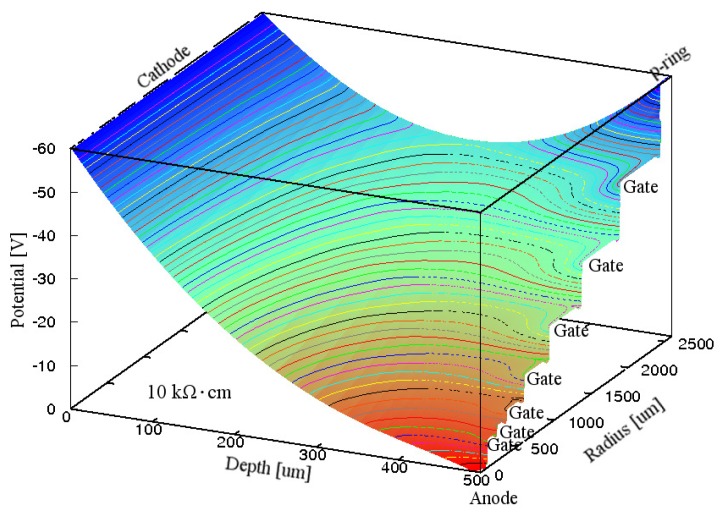
Simulated electric potential distribution in the Si substrate inside the p-ring of a 0.5-mm-thick GSDD with *R*_chip_ of 3.5 mm and ρ_Si_ of 10 kΩ·cm for Gate A. A reverse bias voltage of −60 V was applied to the cathode, p-ring, and seven gates. *Q*_F_ was assumed to be 3 × 10^10^ cm^−2^. Equipotential lines are shown at 1 V intervals.

Figure 2 shows the simulated electric potential distribution in the Si substrate inside the p-ring of the GSDD at *V*_R_ of −60 V for Gate A. The voltage midway between the p-ring and the cathode was −37 V, and the electric field along the electric potential valley was strong enough to make all the electrons produced by an X-ray photon flow smoothly to the anode. Therefore, the electrons produced within the radius of the inner edge of the p-ring can be directed to the anode, indicating that the effective active area is approximately 18 mm^2^.

We fabricated GSDDs using the design of Gate A. In the GSDD, an energy resolution of 145 eV at 5.9 keV was obtained from a ^55^Fe source at −38 °C [41]. The effective active area of the detector was found to be approximately 18 mm^2^ by irradiating X-ray photons through a pinhole with diameter 0.1 mm [40], which is in good agreement with our simulation. These experimental results indicate that GSDDs with the design from which the simulated electric potential distribution similar to that in Figure 2 is obtained can work well.

### 4.2. 0.5-mm-Thick GSDD Formed from a 2-kΩ·cm Si Wafer

**Figure 3:**
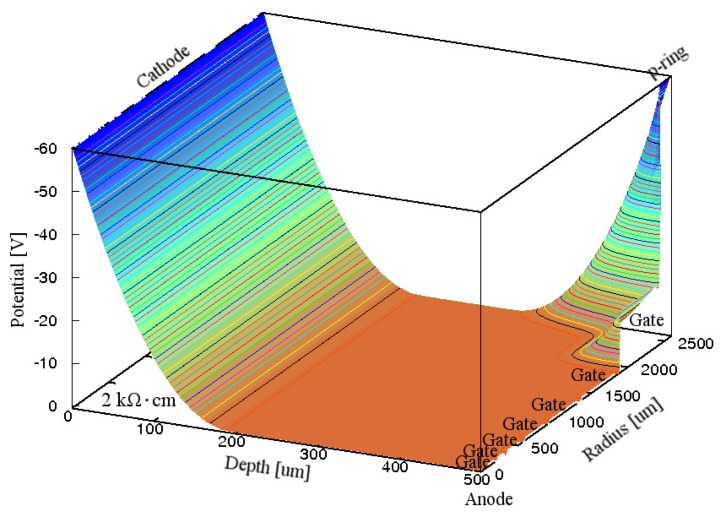
Simulated electric potential distribution in the Si substrate inside the p-ring of a 0.5-mm-thick GSDD with *R*_chip_ of 3.5 mm and ρ_Si_ of 2 kΩ·cm for Gate A. A reverse bias voltage of −60 V was applied to the cathode, p-ring, and seven gates. Equipotential lines are shown at 1 V intervals.

The value of ρ_Si_ was decreased from 10 kΩ·cm to 2 kΩ·cm. Figure 3 shows the simulated electric potential distribution in the Si substrate inside the p-ring of the GSDD with Gate A at *V*_R_ of −60 V. Because the voltage drops at *G*_67_ and *G*_7p_ were too large, the electric potential was almost zero between the anode and the outermost gate, and also over approximately 60% of the n^−^ Si substrate, where the electrons produced by an X-ray photon are recombined with the holes produced by the X-ray photon.

To deplete the whole n^−^ Si substrate,*V*_R_ was increased from −60 to −300 V, following the relation

(1) $$V_{\text{R}}\propto \frac{1}{\rho_{\text{Si}}}$$

**Figure 4:**
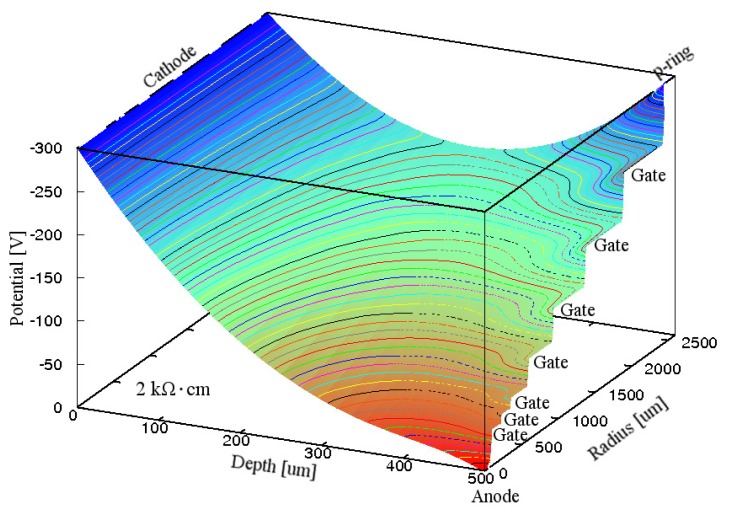
Simulated electric potential distribution in the Si substrate inside the p-ring of a 0.5-mm-thick GSDD with *R*_chip_ of 3.5 mm and ρ_Si_ of 2 kΩ·cm for Gate A. A reverse bias voltage of −300 V was applied to the cathode, p-ring and seven gates. Equipotential lines are shown at 5 V intervals.

Figure 4 shows the simulated electric potential distribution in the Si substrate inside the p-ring of the GSDD with Gate A at *V*_R_ of −300 V. It is clear from Figure 4 that the whole Si substrate was depleted, and all the electrons produced by an X-ray photon flowed smoothly to the anode. This finding indicates that GSDDs with Gate A can work well for any Si resistivity if *V*_R_ follows Equation (1).

*V*_R_ of −300 V was twice that of a commercial 0.5-mm-thick SDD using a 2-kΩ·cm Si wafer. Therefore, a gate pattern that can reduce *V*_R_ was investigated. Because in Figure 3 the voltage decreases at *G*_67_ and *G*_7p_ is too large, *G*_67_ and *G*_7p_ in Gate B were decreased from 0.05 to 0.02 mm. The *G*_34_, *G*_45_ and G56 values were also decreased from 0.05 to 0.02 mm, and *G*_23_ was decreased from 0.03 to 0.02 mm. The value of *G*_a1_ was increased from 0.04 to 0.07 mm, so that the potential at the innermost gate could be increased and the potential around the anode would not be zero. To keep *R*_c_ in Gate B the same as *R*_c_ in Gate A, the values of *W*_3_, *W*_4_, *W*_5_, *W*_6_ and *W*_7_ were changed to 0.21, 0.31, 0.41, 0.51 and 0.54 mm, respectively.

**Figure 5:**
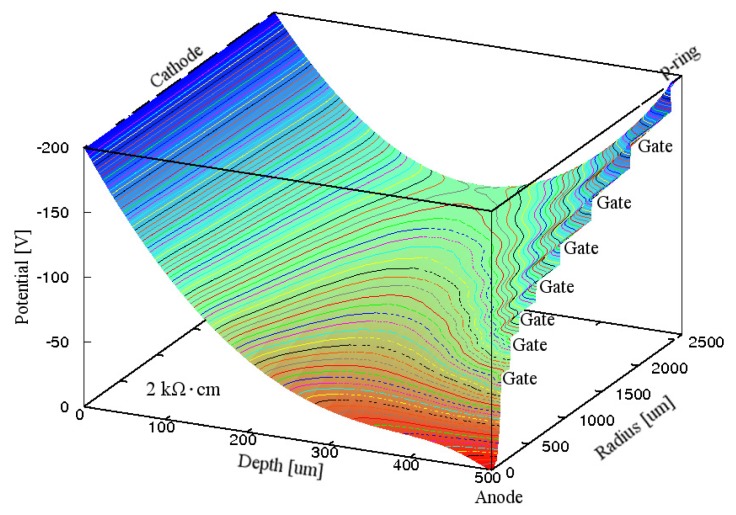
Simulated electric potential distribution in the Si substrate inside the p-ring of a 0.5-mm-thick GSDD with *R*_chip_ of 3.5 mm and ρ_Si_ of 2 kΩ·cm for Gate B. A reverse bias voltage of −200 V was applied to the cathode, p-ring and seven gates. Equipotential lines are shown at 2.5 V intervals.

Figure 5 shows the simulated electric potential distribution in the Si substrate inside the p-ring of the GSDD with Gate B at *V*_R_ of −200 V. Because the voltage midway between the p-ring and the cathode was −92 V, the electric field along the electric potential valley was strong enough to make all the electrons produced by the X-ray photons flow smoothly to the anode.

To reduce *V*_R_ from 200 to 150 V, which is *V*_R_ of commercial 0.5-mm-thick SDDs using 2-kΩ·cm Si wafers, in Gate C the values of *G*_a1_, *G*_12_, *G*_23_, *G*_34_, *G*_45_, *G*_56_, *G*_67_ and *G*_7p_ were changed to 0.11, 0.02, 0.01, 0.01, 0.01, 0.01, 0.005 and 0.005 mm, respectively. To keep *R*_c_ in Gate C the same as *R*_c_ in Gate A, the values of *W*_1_, *W*_2_, *W*_3_, *W*_4_, *W*_5_, *W*_6_ and *W*_7_ were 0.01, 0.05, 0.24, 0.34, 0.44, 0.54 and 0.60 mm, respectively.

**Figure 6:**
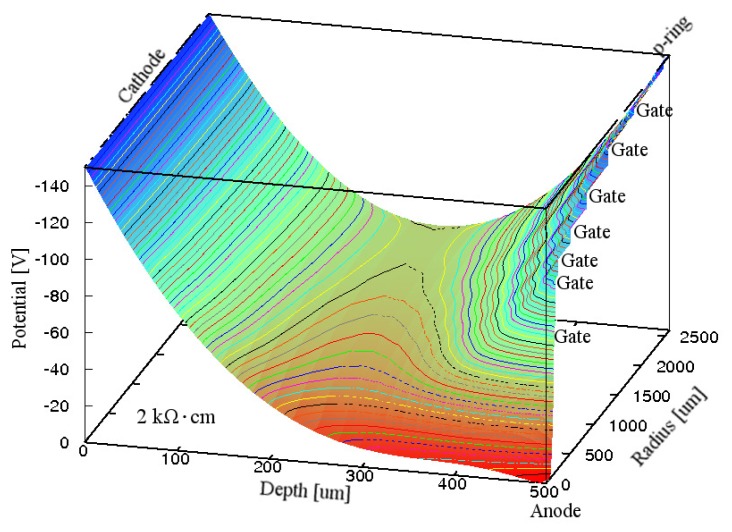
Simulated electric potential distribution in the Si substrate inside the p-ring of a 0.5-mm-thick GSDD with *R*_chip_ of 3.5 mm and ρ_Si_ of 2 kΩ·cm for Gate C. A reverse bias voltage of −150 V was applied to the cathode, p-ring, and seven gates. Equipotential lines are shown at 2.5 V intervals.

Figure 6 shows the simulated electric potential distribution in the Si substrate inside the p-ring of the GSDD for Gate C at *V*_R_ of −150 V. In the electric potential distribution, the voltage midway between the p-ring and the cathode was approximately −78 V, and consequently the electric field along the electric potential valley strong enough to make all the electrons produced by the X-ray photons flow smoothly to the anode.

### 4.3. 1-mm-Thick GSDD Formed from a 2-kΩ·cm Si Wafer

To detect traces of hazardous or radioactive elements in food, soil, and the human body effectively, the absorption of X-ray fluorescence photons of these elements, such as Cd (23.1 keV) and Cs (30.8 keV), by GSDDs must be increased. However, the thickness of the Si substrates in commercial SDDs is approximately 0.5 mm; thus, the absorbed fractions of Cd and Cs X-ray fluorescence photons are 29.1% and 14.4%, respectively. In contrast, for a 1-mm-thick Si substrate, the absorbed fractions increase to 49.7% and 26.8%, respectively. In other words, the commercial SSDs up to 0.5 mm thick can detect photons with energies up to 27 keV for X-ray absorbance higher than 20%, whereas our gate pattern for the GSDD can detect photons with energies up to 35 keV. Here, we simulate the electric potential distribution in the GSDD with a Si thickness of 1 mm.

In the 1-mm-thick GSDDs, *d*_g_ was changed from 0.75 to 3 µm to avoid SiO_2_ breakdown caused by the high electric field. In Gate D, the values of *G*_a1_, *G*_12_, *G*_23_, *G*_34_, *G*_45_, *G*_56_, *G*_67_ and *G*_7p_ were changed to 0.33, 0.06, 0.02, 0.02, 0.02, 0.02, 0.01 and 0.01 mm, respectively. To keep *R*_c_ in Gate D the same as *R*_c_ in Gate A, the values of *W*_1_, *W*_2_, *W*_3_, *W*_4_, *W*_5_, *W*_6_ and *W*_7_ were changed to 0.02, 0.07, 0.18, 0.28, 0.38, 0.47 and 0.51 mm, respectively.

To deplete the whole n^−^ Si substrate, the value of *V*_R_ was increased from 150 to 600 V, following the relation

(2) $$V_{\text{R}}\propto d_{\text{Si}}^{2}$$

**Figure 7:**
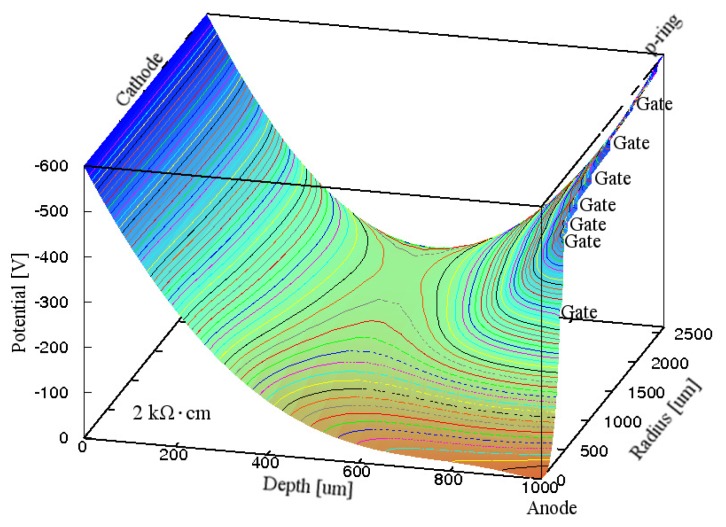
Simulated electric potential distributions in the Si substrate inside the p-ring of a 1-mm-thick GSDD with *R*_chip_ of 3.5 mm and ρ_Si_ of 2 kΩ·cm for Gate D. A reverse bias voltage of −600 V was applied to the cathode, p-ring, and seven gates. Equipotential lines are shown at 10 V intervals.

Figure 7 shows the simulated electric potential distribution for Gate D in the Si substrate inside the p-ring of the GSDD at *V*_R_ of −600 V. The voltage at the saddleback was approximately −175 V. Because the average electric field toward the anode along the electric potential valley was approximately 950 V/cm, the average electron drift velocity was higher than 1 × 10^6^ cm/s at the operating temperature (≤0 °C). This was caused by the electron mobility of 1450 cm^2^- V^−1^·s^−1^ in the Si substrate at room temperature [44]. This indicates that the electric field along the electric potential valley was strong enough to make all the electrons produced by the X-ray photons flow smoothly to the anode.

For a Si pin diode with *d*_Si_ of 1 mm and ρ_Si_ of 2 kΩ·cm, a reverse bias of approximately −1500 V is required to deplete the whole Si layer. However, for the GSDD, a reverse bias of only −600 V was required, which is an advantage of GSDDs.

## 5. Conclusions

GSDDs are inexpensive Si X-ray detectors because of their simple structure. Although we have investigated GSDDs with 10-kΩ·cm Si because we have designed thicker GSDDs to detect X-ray photons with high energies, 10-kΩ·cm Si wafers are much more expensive than 2-kΩ·cm Si wafers, from which commercial SDDs are fabricated. Therefore, GSDDs with 2-kΩ·cm Si were investigated to develop low-cost X-ray detectors that can accurately detect photon counts and energies of X-ray fluorescence photons with energies of up to 35 keV. Device simulations of GSDDs with 0.5- and 1-mm-thick, 2-kΩ·cm Si substrates indicated that the X-ray detectors should work well when they are produced by using current fabrication processes. GSDDs with one gate pattern can work well for any resistivity Si substrate if the reverse bias is inversely proportional to the resistivity of the Si substrate. These findings indicate that the cost of portable X-ray fluorescence instruments can be reduced considerably.
